# Supplementary material for: Mitigating the non-specific uptake of immunomagnetic microparticles enables the extraction of endothelium from human fat
Source: Commun Biol. 2021 Oct 20;4:1205. doi: 10.1038/s42003-021-02732-8 (PMC8528810; doi:10.1038/s42003-021-02732-8)
Supplement: Supplementary file 2 — Supplementary Information [file 42003_2021_2732_MOESM2_ESM.pdf]

Mitigating the non-specific uptake of immunomagnetic microparticles enables the extraction of endothelium from human fat

Supplementary Information

Jeremy A. Antonyshyn[1,2], Vienna Mazzoli[1,2], Meghan J. McFadden[1,2], Anthony O. Gramolini[2,3], Stefan O.P. Hofer[4,5], Craig A. Simmons[1,2,6], J. Paul Santerre[1,2,7*]

[1]Institute of Biomedical Engineering, University of Toronto, Toronto, ON, Canada; [2]Translational Biology and Engineering Program, Ted Rogers Centre for Heart Research, Toronto, ON, Canada; [3]Department of Physiology, University of Toronto, Toronto, ON, Canada; [4]Division of Plastic, Reconstructive, and Aesthetic Surgery, University of Toronto, Toronto, ON, Canada; [5]Departments of Surgery and Surgical Oncology, University Health Network, Toronto, ON, Canada; [6]Department of Mechanical and Industrial Engineering, University of Toronto, Toronto, ON, Canada; [7]Faculty of Dentistry, University of Toronto, Toronto, ON, Canada

[*]*Corresponding Author:* J. Paul Santerre
paul.santerre@utoronto.ca

Supplementary Tables and Figures

Supplementary Table 1. Nucleotide sequences of the primers used for the reverse transcription quantitative real-time polymerase chain reaction assays. *GAPDH* represents glyceraldehyde-3-phosphate dehydrogenase; *CDH5*, cadherin-5 (vascular endothelial -cadherin, VE-cadherin); and, *VWF*, von Willebrand Factor (vWF).

Gene	Forward Primer<br>*5' → 3'*	Reverse Primer<br>*3' → 5'*
GAPDH	CTCCTGTTCGACAGTCAGCC	CCTCAGTTGCCTAAACCAGC
PECAM1	GTCCCTGATGCCGTGGAAAG	AATACTTGGACGGGACGAGG
CDH5	CTTCACCCAGACCAAGTACACA	TGGTCCTGCGAAAGTGGTAA
VWF	TTGACGGGGAGGTGAATGTG	GCACCAGGACTTCGTCTGTA

Supplementary Table 2. Size distributions of the immunomagnetic microparticles (IMPs). IMPs are labeled on the basis of their target antigen and modal diameter. Their size distributions were evaluated using a Coulter counter, collecting data for a modal count of 5,000. cIMP represents cleavable IMPs; N, number of IMPs; Std Dev, standard deviation; and, PDI, polydispersity index.

IMP	N	Mean (µm)	Median (µm)	Mode (µm)	Std Dev (µm)	PDI
0.9 µm Anti-CD31 IMPs	249,500	1.26	1.22	0.93	0.34	0.071
3.9 µm Anti-CD31 IMPs	217,400	5.07	4.89	3.91	1.24	0.060
4.4 µm Anti-CD31 IMPs	59,312	4.59	4.52	4.44	0.61	0.018
4.8 µm Anti-CD31 cIMPs	62,669	4.95	4.89	4.81	0.56	0.013
4.8 µm Anti-CD93 cIMPs	60,948	4.96	4.89	4.84	0.54	0.012
8.7 µm Anti-CD31 IMPs	60,592	8.84	8.71	8.65	1.50	0.029

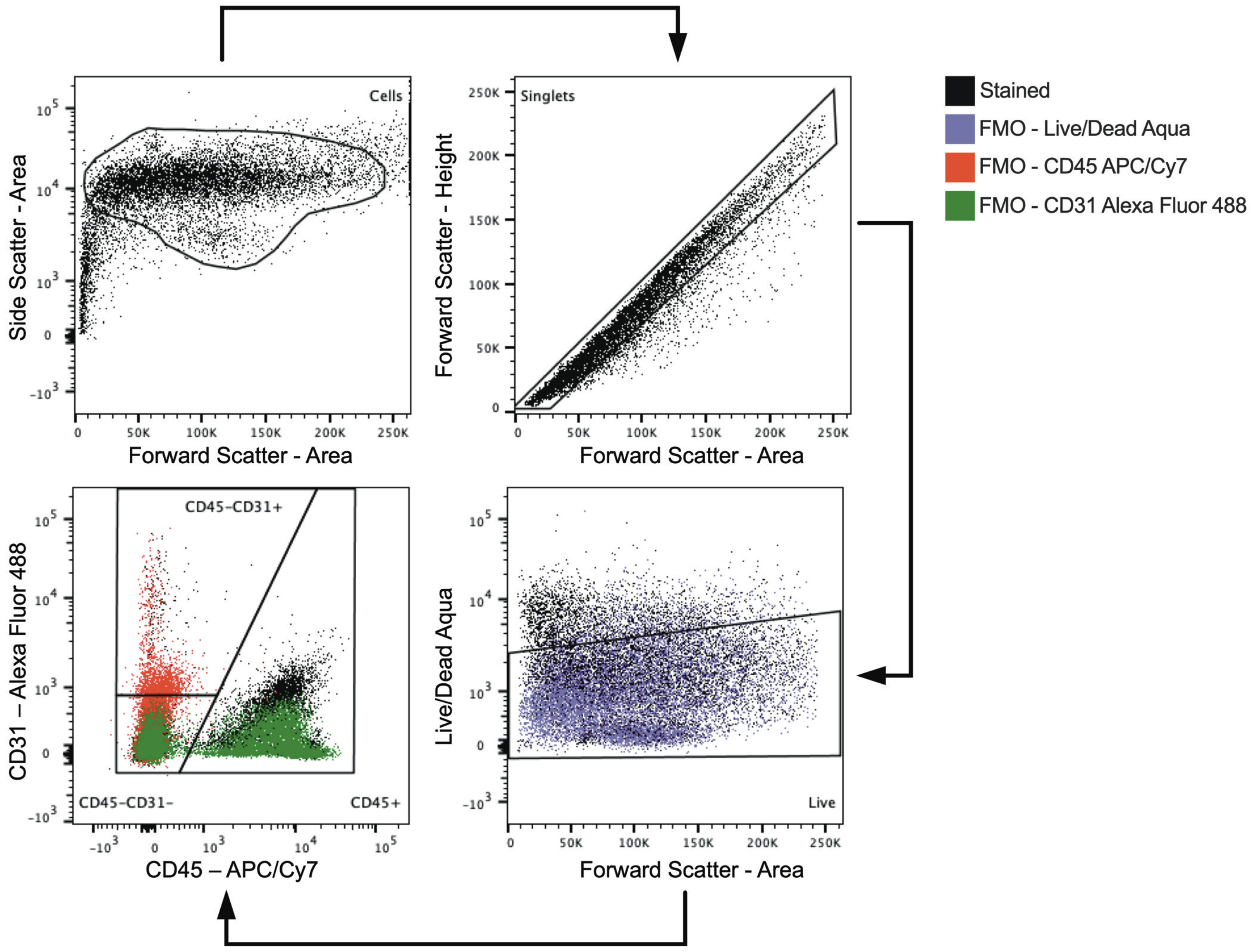

Supplementary Figure 1. Gating strategy used to characterize the composition of the stromal vascular fraction of enzymatically digested human subcutaneous abdominal white adipose tissue and the purity of its primary cultures. Gates were set using fluorescence minus one (FMO) controls where applicable.

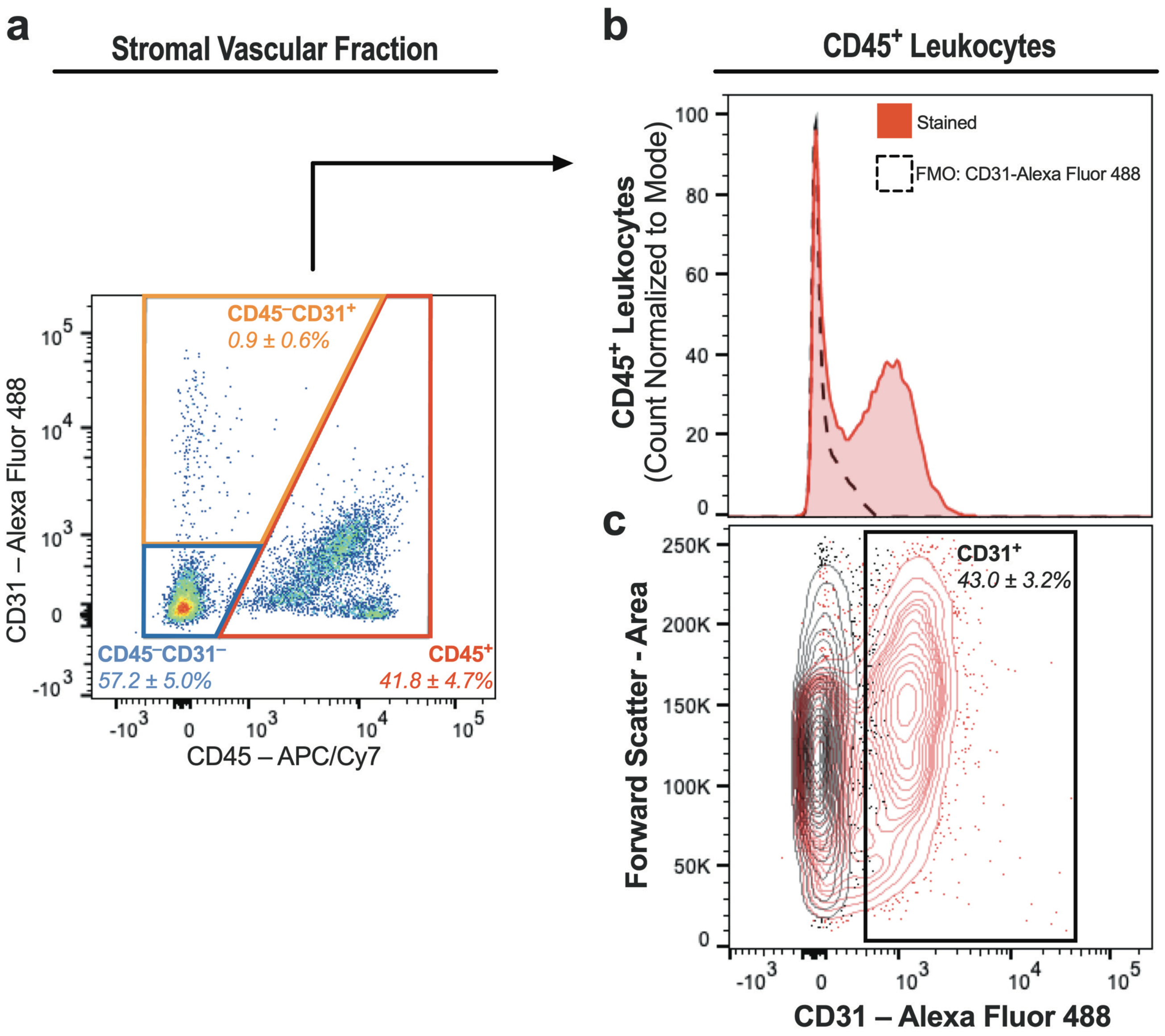

Supplementary Figure 2. Cell-surface expression of the characteristic endothelial marker CD31 by CD45⁺ leukocytes in the stromal vascular fraction of enzymatically digested human subcutaneous abdominal white adipose tissue. **(a)** Pseudocolour plot depicting the expression of CD45 and CD31 by stromal vascular cells, as well as a **(b)** histogram and **(c)** contour plot depicting the co-expression of CD31 by the CD45⁺ leukocytes. FMO represents 'fluorescence minus one' controls. Values represent mean ± standard deviation. This experiment was performed in biological triplicate, using cells derived from three different donors (n = 3 biologically independent samples).

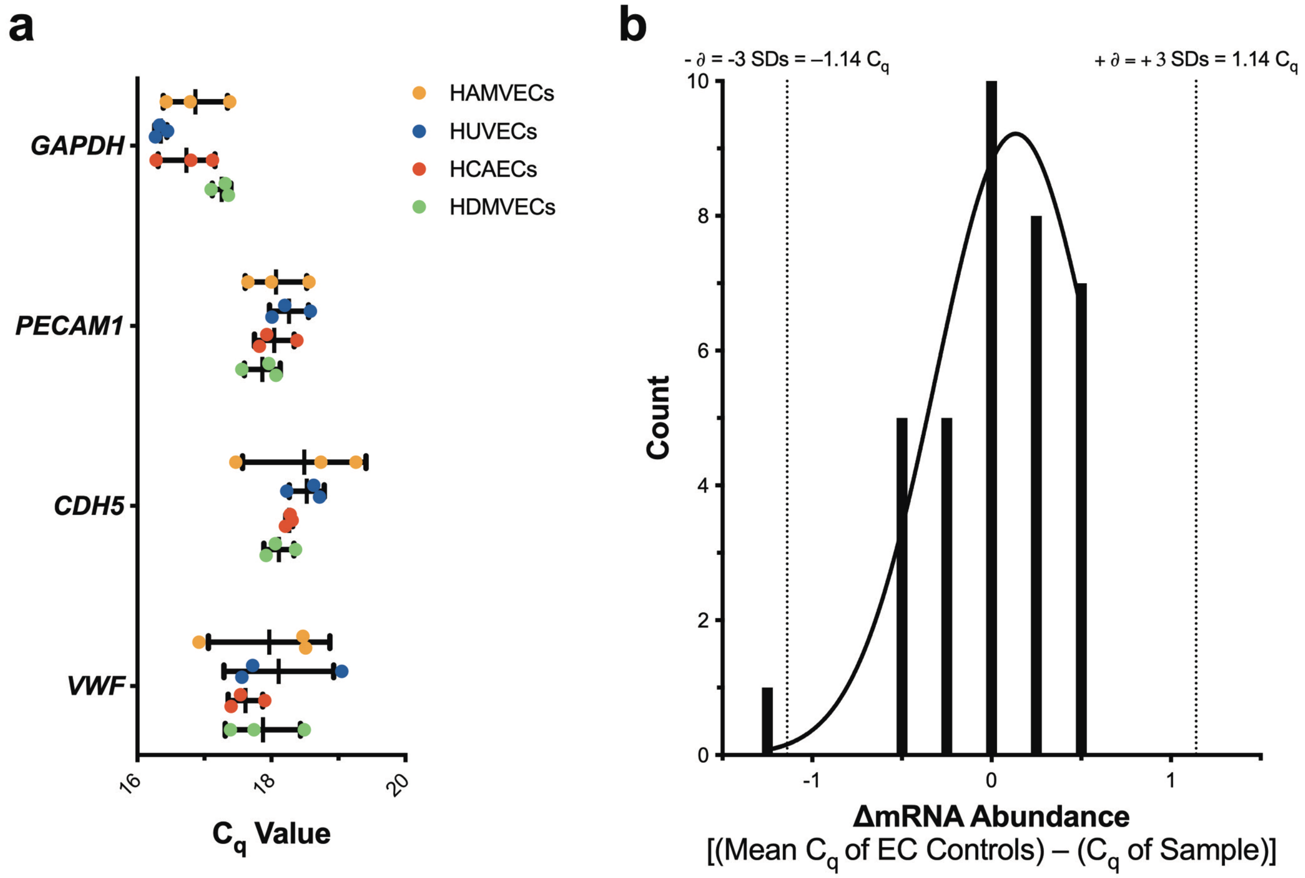

Supplementary Figure 3. Abundance of transcripts encoding *PECAM1*, *CDH5*, and *VWF* in endothelial cells (ECs). Human adipose tissue-derived microvascular ECs (HAMVECs) were compared with EC controls representative of the predominant endothelial specializations, namely human umbilical vein ECs (HUVECs; macrovascular, venous endothelium), human coronary artery ECs (HCAECs; macrovascular, arterial endothelium), and human dermal microvascular ECs (HDMVECs; microvascular endothelium). **(a)** The abundance of messenger ribonucleic acid (mRNA) encoding *GAPDH*, *PECAM1*, *CDH5*, and *VWF* was evaluated by reverse transcription quantitative real-time polymerase chain reaction, and **(b)** the equivalence margin (∂) was set to three standard deviations (SDs) of the Gaussian distribution of quantification cycle (C_q) values about their gene-normalized means for the two one-sided test for equivalence. The mRNA abundances detected in HAMVECs are shown for reference **(a)**, but were not included in the determination of the equivalence margin **(b)**. Values represent mean ± standard deviation. This experiment was performed in biological triplicate, using cells derived from three different donors (n = 3 biologically independent samples).

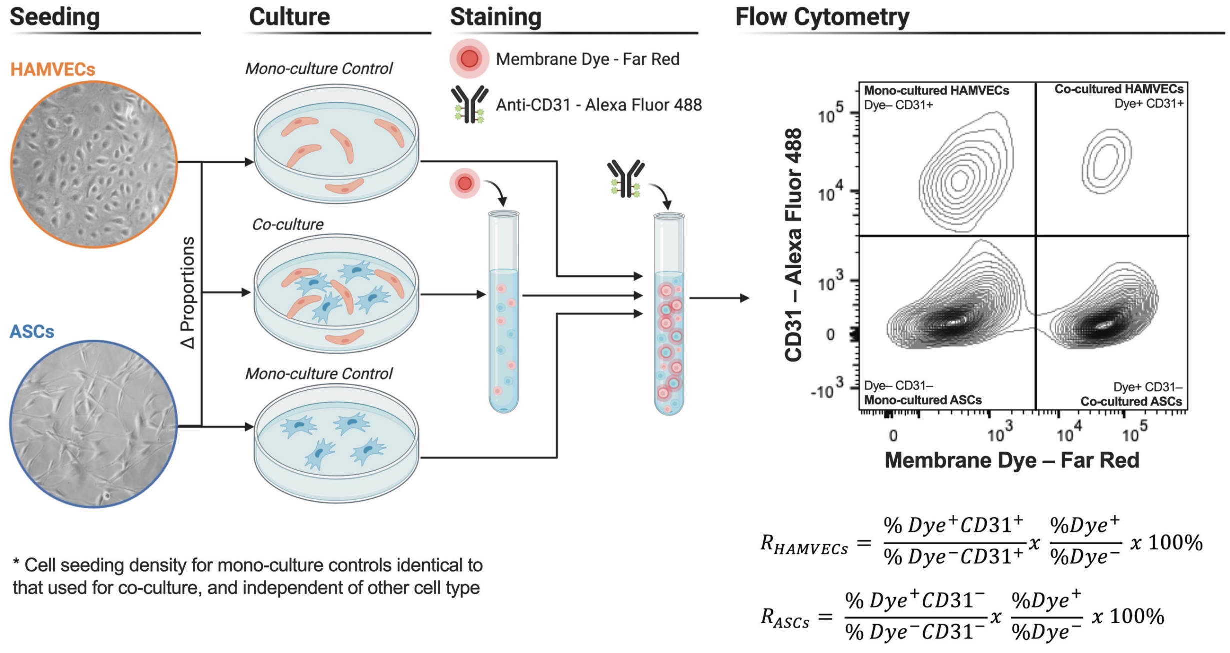

Supplementary Figure 4. Schematic depicting the evaluation of population growth rates of human adipose tissue-derived microvascular endothelial cells (HAMVECs) and adipose tissue-derived stromal/stem cells (ASCs) in co-culture. HAMVECs and ASCs were seeded together in pre-defined proportions, or separately at their respective densities. Cells were cultured for 4 days, after which co-cultured cells were stained with a membrane dye and combined with the mono-cultured controls. The mixture was then stained anti-human CD31-Alexa Fluor 488 antibodies and analyzed by flow cytometry. Population growth rates in co-culture were calculated using the depicted formulas, where $R_{HAMVECs}$ and R_{ASCs} represent the population growth rates of HAMVECs and ASCs relative to their mono-cultured controls, respectively. Created with BioRender.com

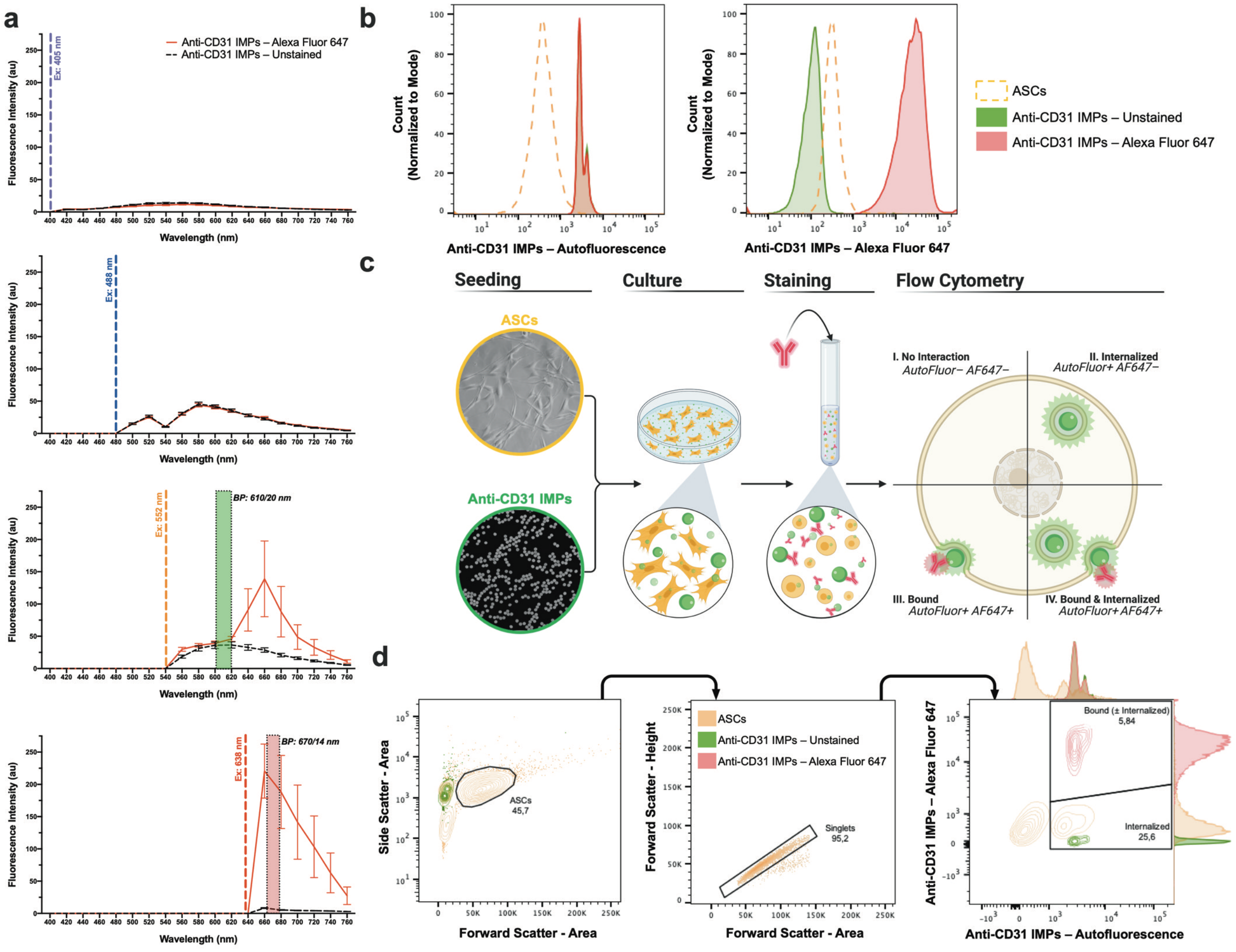

Supplementary Figure 5. Development and validation of the immunomagnetic microparticle (IMP) uptake and localization assay. IMPs were detected on the basis of their autofluorescence, and a membrane-impermeable secondary antibody directed against their binding moiety was used to discriminate between their extracellular and intracellular localization. **(a)** Representative emission spectra of IMPs with and without being stained with a goat anti-mouse IgG-Alexa Fluor 647 secondary antibody. Values represent mean ± standard deviation of 30 IMPs. **(b)** Validation of candidate excitation-emission characteristics of the IMPs by flow cytometry. An excitation (Ex) wavelength of 552 nm and a 610/20 nm emission (Em) bandpass filter (BP) was used to detect the autofluorescent IMPs (Ex: 552 nm; Em: 610/20 nm), and an excitation wavelength of 638 nm and a 670/14 nm emission BP was used to detect IMPs conjugated with the Alexa Fluor 647 secondary antibody (Ex: 638 nm; Em: 670/14 nm). The autofluorescence of adipose tissue-derived stromal/stem cells (ASCs) in both channels is shown for reference. **(c)** Schematic depicting the IMP binding and internalization assay. ASCs were exposed to IMPs for pre-defined durations before being stained with a goat anti-mouse IgG-Alexa Fluor 647 secondary antibody and analyzed by flow cytometry. Created with BioRender.com **(d)** Gating strategy used to discriminate cells from free IMPs, IMP-laden ASCs from IMP-free ASCs, and a membrane-bound IMP localization from an intracellular IMP localization.

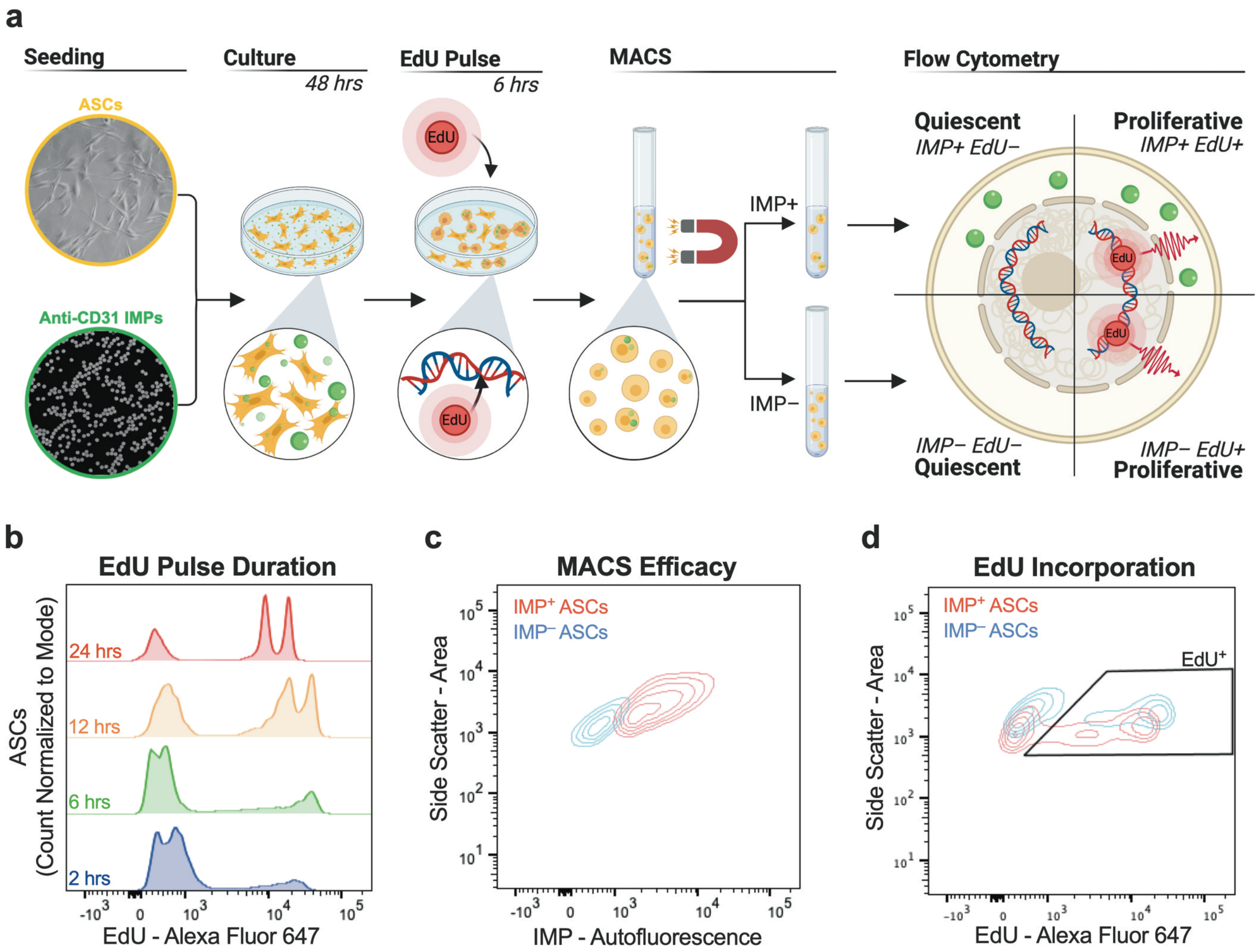

Supplementary Figure 6. Deoxyribonucleic acid (DNA) synthesis by immunomagnetic microparticle (IMP)-laden and IMP-free adipose tissue-derived stromal/stem cells (ASCs) was assessed based on their incorporation of the thymidine analogue 5-ethynyl-2′-deoxyuridine (EdU). **(a)** Schematic depicting the EdU incorporation assay. ASCs were cultured with anti-CD31 IMPs for 48 hr, before being rinsed with phosphate-buffered saline to remove free IMPs and pulsed with EdU for 6 hr. IMP-laden ASCs were then magnetically separated from IMP-free ASCs before being stained and analyzed by flow cytometry. Created with BioRender.com **(b)** Determination of the EdU pulse duration. EdU pulses 6 hr in duration labeled cells in the S phase of the cell cycle, while pulses ≥ 12 hr yielded bimodal EdU^{+} populations indicative of ≥ 2 rounds of mitosis. **(c)** Greater autofluorescence of IMP-laden ASCs when compared with IMP-free ASCs supports the efficacy of the magnet-assisted cell sorting. Loss in resolution between IMP^{+} and IMP^{-} ASCs due to different fixation and permeabilization procedure required for EdU staining. **(d)** Representative contour plot depicting the incorporation of EdU by IMP-laden and IMP-free ASCs.

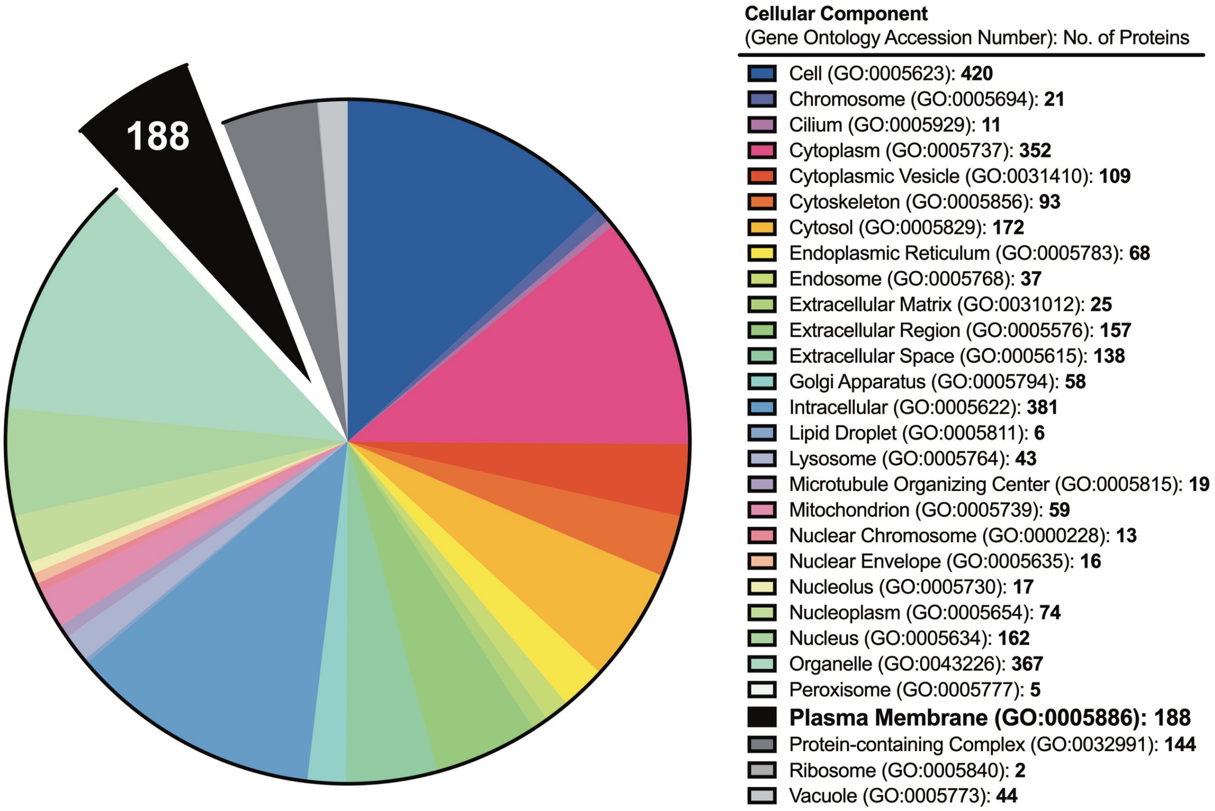

Supplementary Figure 7. Gene ontological mapping of the 457 proteins enriched in human adipose tissue-derived microvascular endothelial cells (HAMVECs) compared with adipose tissue-derived stromal/stem cells (ASCs) to their cellular components identified 188 that were localized to the plasma membrane.

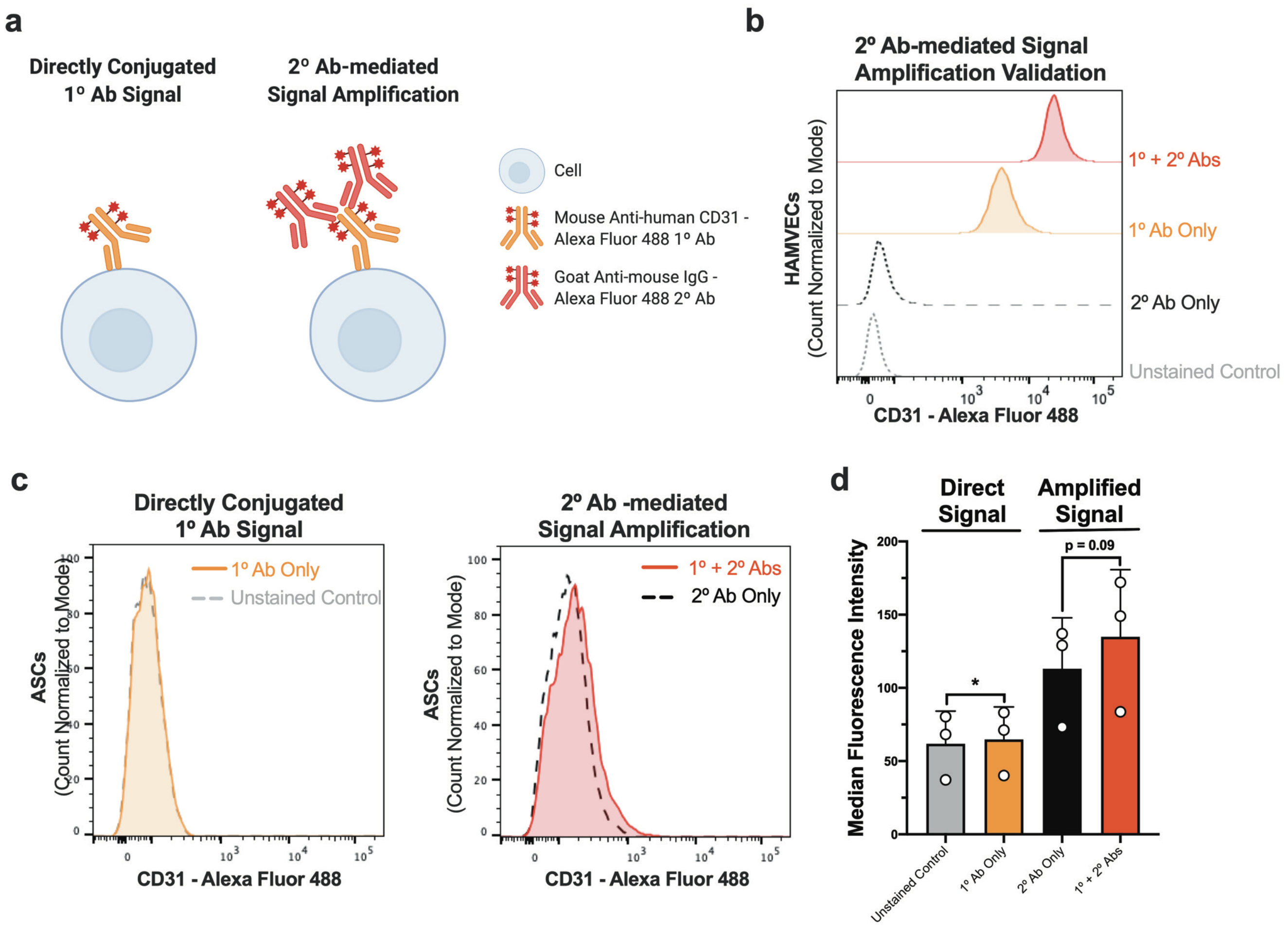

Supplementary Figure 8. Secondary antibody -mediated signal amplification supports the cell-surface expression of CD31 by adipose tissue-derived stromal/stem cells (ASCs). **(a)** Schematic depicting the principle underlying secondary antibody -mediated signal amplification. Created with BioRender.com **(b)** Validation of secondary antibody -mediated signal amplification in human adipose tissue-derived microvascular endothelial cells (HAMVECs). **(c)** Representative histograms depicting the detection of cell-surface CD31 expression by ASCs using Alexa Fluor 488-conjugated anti-human CD31 antibodies with and without their secondary antibody -mediated signal amplification, and **(d)** their respective median fluorescence intensities. Values represent mean ± standard deviation. *p < 0.05. 1º Ab represents primary antibody; and, 2º Ab, secondary antibody. This experiment was performed in biological triplicate, using cells derived from three different donors (n = 3 biologically independent samples).

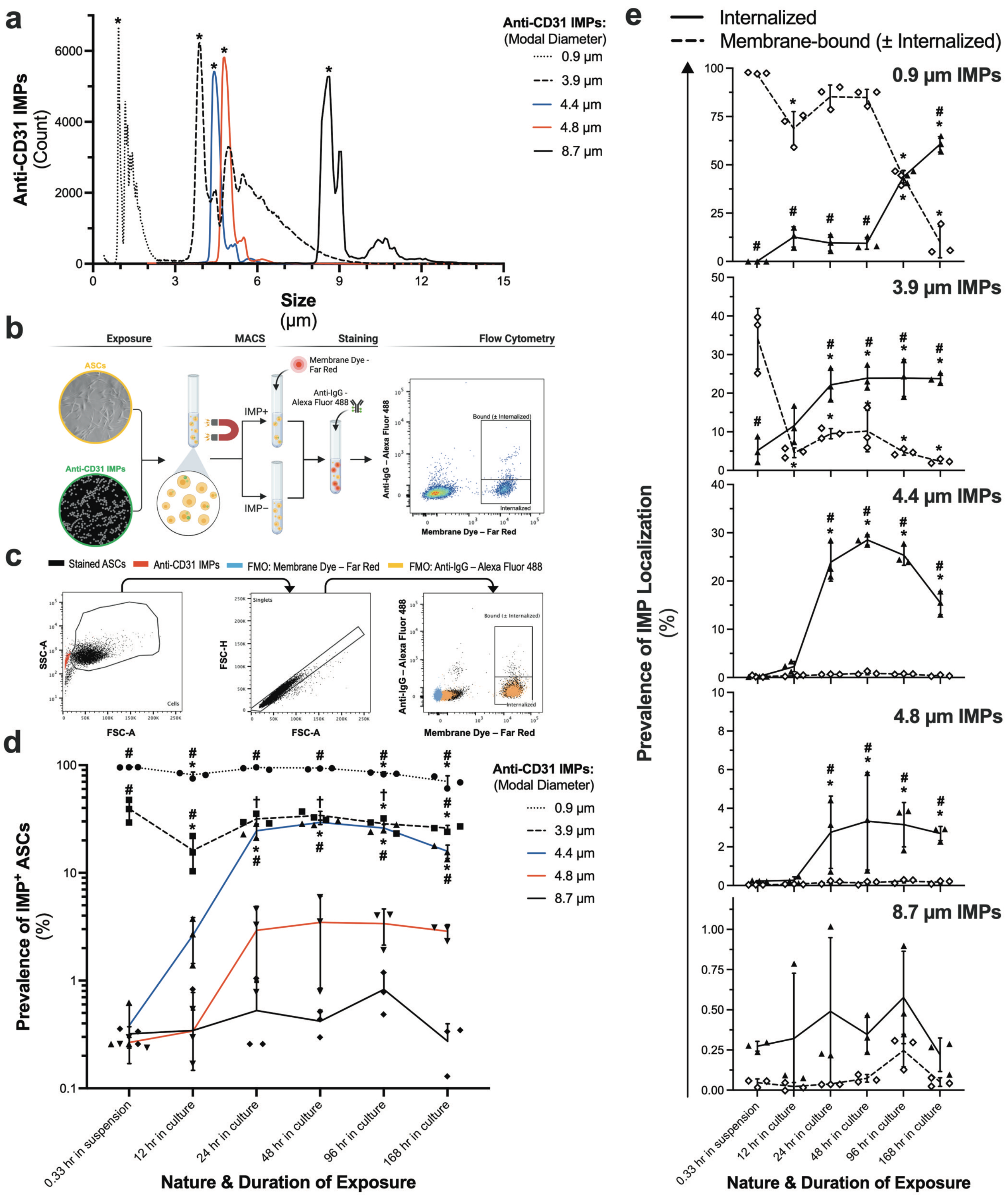

Supplementary Figure 9. Size affects the extent and temporal dynamics underlying the uptake of immunomagnetic microparticles (IMPs) by adipose tissue-derived stromal/stem cells (ASCs). (**a**) Size distributions of five distinct anti-CD31 IMPs. *$p < 0.05$ compared with all other IMPs. (**b**) Schematic de-

picting the IMP uptake assay. ASCs were exposed to the different anti-CD31 IMPs for 20 min in suspension or different durations in culture before using magnet-assisted cell sorting (MACS) to separate IMP^+ ASCs from $IMP^–$ ASCs. IMP^+ ASCs were stained with a membrane dye before being recombined with the $IMP^–$ ASCs. The mixture was then stained with a membrane-impermeable secondary antibody directed against the binding moiety of the IMPs to discriminate between their intracellular and extracellular localization. The mixture was assessed by flow cytometry. Created with BioRender.com (**c**) Gating strategy used to assess the prevalence of IMP uptake by ASCs and their localization by flow cytometry. FSC-A represents forward scatter area; SSC-A, side scatter area; FSC-H, forward scatter height; FMO, fluorescence minus one controls. (**d**) Total uptake (i.e. membrane-bound and/or internalized) of the different anti-CD31 IMPs by ASCs after 20 min in suspension (i.e. labeling conditions for MACS) and different durations in culture. Values represent mean ± standard deviation; $^*p < 0.05$ relative to 20 min in suspension; $^{\#}p < 0.05$ relative to all smaller IMPs at the same time-point; and, $^{\dagger}p < 0.05$ relative to IMPs with a modal diameter of 4.8 µm and 8.7 µm. (**e**) Prevalence of IMP localization after 20 min in suspension (i.e. labeling conditions for MACS) and different durations in culture. $^*p < 0.05$ compared with respective localization in suspension; $^{\#}p < 0.05$ compared with membrane-bound localization at the same time-point. Experiments were performed in biological triplicate, using cells derived from three different donors (n = 3 biologically independent samples).

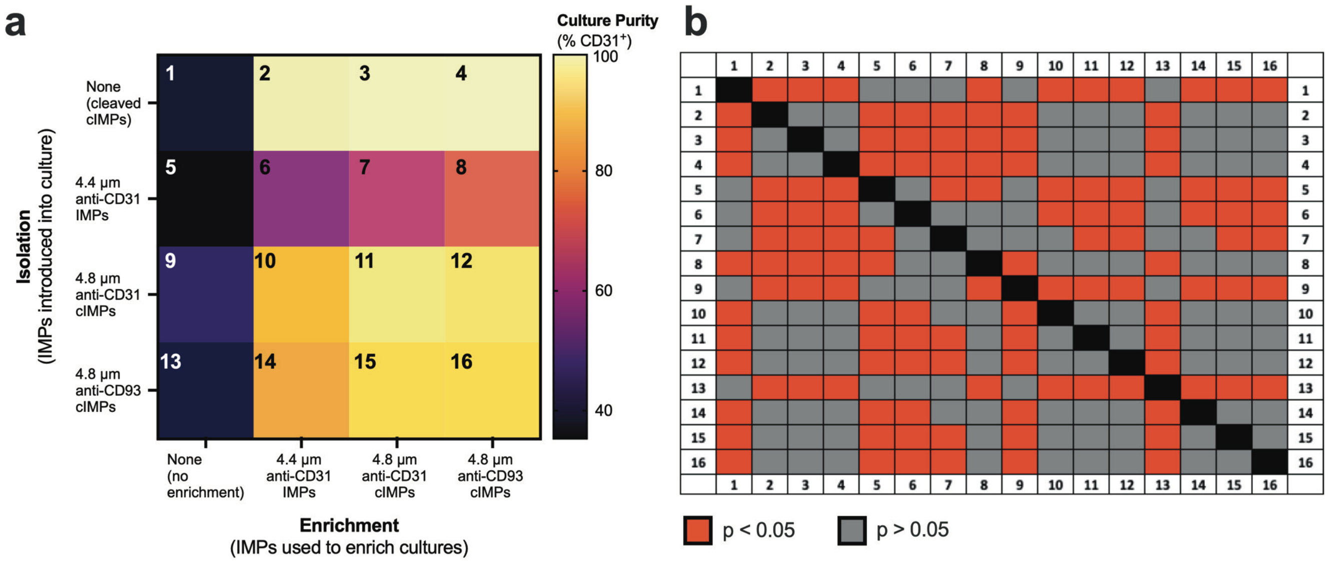

Supplementary Figure 10. Effects of alternative target antigens (CD31 vs. CD93), sizes (4.4 µm vs. 4.8 µm), and exposures (introduced vs. excluded from culture) of immunomagnetic microparticles (IMPs) on the enrichment efficacy of the magnet-assisted cell sorting procedure elucidated using an *in vitro* model of contaminated primary cultures. Anti-CD31 IMPs are 4.4 µm in diameter, and anti-CD31 and anti-CD93 cleavable (c)IMPs are 4.8 µm in diameter. Shown are heat maps depicting **(a)** the mean purity of the different cultures, and **(b)** the results of the corresponding statistical comparisons. This experiment was performed in biological triplicate, using cells derived from three different donors (n = 3 biologically independent samples).
