# Supplementary material for: Mitigating the non-specific uptake of immunomagnetic microparticles enables the extraction of endothelium from human fat
Source: Commun Biol. 2021 Oct 20;4:1205. doi: 10.1038/s42003-021-02732-8 (PMC8528810; doi:10.1038/s42003-021-02732-8)
Supplement: Supplementary file 3 — Description of Supplementary Files [file 42003_2021_2732_MOESM3_ESM.pdf]

Description of Additional Supplementary Files

File name: Supplementary Data 1
Description: Proteomic comparison of human adipose tissue-derived microvascular endothelial cells (HAMVECs) with endothelial cell (EC) controls representative of the predominant endothelial specializations, namely human umbilical vein ECs (HUVECs; macrovascular, venous), human coronary artery ECs (HCAECs; macrovascular, arterial), and human dermal microvascular ECs (HDMVECs; microvascular).

File name: Supplementary Data 2
Description: Proteomic comparison of human adipose tissue-derived microvascular endothelial cells (HAMVECs) and adipose tissue-derived stromal/stem cells (ASCs) by liquid chromatography tandem mass spectrometry.

File name: Supplementary Data 3
Description: Source data underlying the graphs and charts presented in the main figures of the manuscript.
