# Supplementary material for: Mitigating the non-specific uptake of immunomagnetic microparticles enables the extraction of endothelium from human fat
Source: Commun Biol. 2021 Oct 20;4:1205. doi: 10.1038/s42003-021-02732-8 (PMC8528810; doi:10.1038/s42003-021-02732-8)
Supplement: Supplementary file 7 — Reporting Summary [file 42003_2021_2732_MOESM7_ESM.pdf]

nature research

Corresponding author(s): J. Paul Santerre

Last updated by author(s): Aug 10, 2021

Reporting Summary

Nature Research wishes to improve the reproducibility of the work that we publish. This form provides structure for consistency and transparency in reporting. For further information on Nature Research policies, see our Editorial Policies and the Editorial Policy Checklist.

Statistics

For all statistical analyses, confirm that the following items are present in the figure legend, table legend, main text, or Methods section.

n/a	Confirmed	
☐	☒	The exact sample size (*n*) for each experimental group/condition, given as a discrete number and unit of measurement
☐	☒	A statement on whether measurements were taken from distinct samples or whether the same sample was measured repeatedly
☐	☒	The statistical test(s) used AND whether they are one- or two-sided<br>*Only common tests should be described solely by name; describe more complex techniques in the Methods section.*
☒	☐	A description of all covariates tested
☐	☒	A description of any assumptions or corrections, such as tests of normality and adjustment for multiple comparisons
☐	☒	A full description of the statistical parameters including central tendency (e.g. means) or other basic estimates (e.g. regression coefficient) AND variation (e.g. standard deviation) or associated estimates of uncertainty (e.g. confidence intervals)
☐	☒	For null hypothesis testing, the test statistic (e.g. *F*, *t*, *r*) with confidence intervals, effect sizes, degrees of freedom and *P* value noted<br>*Give P values as exact values whenever suitable.*
☒	☐	For Bayesian analysis, information on the choice of priors and Markov chain Monte Carlo settings
☒	☐	For hierarchical and complex designs, identification of the appropriate level for tests and full reporting of outcomes
☒	☐	Estimates of effect sizes (e.g. Cohen's *d*, Pearson's *r*), indicating how they were calculated

Our web collection on statistics for biologists contains articles on many of the points above.

Software and code

Policy information about availability of computer code

Data collection	BD FACSDiva v8.0.1; Leica Application Suite X v3.5.5.19976; Tune v2.8; Xcalibur v4.0.27.19; Multisizer 4e v4.03; EnVision Manager software version 1.14.3049.528
Data analysis	FlowJo v10.7.1; Bio-Rad CFX Maestro 1.1 v4.1.2433.1219; Fiji v2.1.0/1.53c; MaxQuant v1.6.0.1; Perseus v1.6.1.2; PANTHER v15.0; GO Term Mapper; Prism 8 v8.4.3

For manuscripts utilizing custom algorithms or software that are central to the research but not yet described in published literature, software must be made available to editors and reviewers. We strongly encourage code deposition in a community repository (e.g. GitHub). See the Nature Research guidelines for submitting code & software for further information.

Data

Policy information about availability of data

All manuscripts must include a data availability statement. This statement should provide the following information, where applicable:

- Accession codes, unique identifiers, or web links for publicly available datasets
- A list of figures that have associated raw data
- A description of any restrictions on data availability

Raw data and search results from the liquid chromatography tandem mass spectrometry are available from the MassIVE repository (accession no. MSV000086982; password: Endothelial), and the corresponding tabulated datasets are supplied in Supplementary Data 1 and 2. Source data underlying the graphs and charts presented in the main figures are provided in Supplementary Data 3. All other data are available from the corresponding author upon reasonable request.

Field-specific reporting

Please select the one below that is the best fit for your research. If you are not sure, read the appropriate sections before making your selection.

[x] Life sciences   [ ] Behavioural & social sciences   [ ] Ecological, evolutionary & environmental sciences

For a reference copy of the document with all sections, see nature.com/documents/nr-reporting-summary-flat.pdf

Life sciences study design

All studies must disclose on these points even when the disclosure is negative.

Sample size	All experiments were performed using cells derived from three different donors, unless indicated otherwise. This was preemptively determined to be the minimum number of biological samples needed to define the central tendency and variation in our in vitro experiments.
Data exclusions	Proteins detected in only one of three biological replicates by LC-MS/MS were excluded from further analyses due to our interest in characterizing the traits of the populations, not individual samples. No other data was excluded.
Replication	All experiments were repeated at least three times, using cells derived from at least three different donors.
Randomization	Randomization was not relevant to this study. Donors were enrolled in this study until three populations of HAMVECs were successfully isolated, which were then used for subsequent experiments to investigate the challenge underlying their acquisition.
Blinding	Blinding was not possible in this study, as the primary objective was to elucidate the challenge underlying stromal cell overgrowth of HAMVECs - actively manipulating, monitoring and discriminating between these two distinct cell types was central to the investigation.

Reporting for specific materials, systems and methods

We require information from authors about some types of materials, experimental systems and methods used in many studies. Here, indicate whether each material, system or method listed is relevant to your study. If you are not sure if a list item applies to your research, read the appropriate section before selecting a response.

Materials & experimental systems

n/a	Involved in the study	
[ ]	[x]	Antibodies
[x]	[ ]	Eukaryotic cell lines
[x]	[ ]	Palaeontology and archaeology
[x]	[ ]	Animals and other organisms
[ ]	[x]	Human research participants
[x]	[ ]	Clinical data
[x]	[ ]	Dual use research of concern

Methods

n/a	Involved in the study	
[x]	[ ]	ChIP-seq
[ ]	[x]	Flow cytometry
[x]	[ ]	MRI-based neuroimaging

Antibodies

Antibodies used	All vendors and corresponding catalogue numbers of antibodies used in this study are delineated in the Methods.
Validation	All antibodies were validated by their respective manufacturers, literature, and data presented in this manuscript.

Human research participants

Policy information about studies involving human research participants

Population characteristics	Subcutaneous abdominal white adipose tissue was obtained with informed consent from patients undergoing reconstructive breast surgery at the University Health Network (Toronto, Ontario, Canada; institutional research ethics board approval no. 13-6437-CE).
Recruitment	Patients presenting to the University Health Network (Toronto, Ontario, Canada) for reconstructive breast surgery were approached for informed consent to participate in this study (institutional research ethics board approval no. 13-6437-CE).
Ethics oversight	University Health Network (Toronto, Ontario, Canada; institutional research ethics board approval no. 13-6437-CE).

Note that full information on the approval of the study protocol must also be provided in the manuscript.

Flow Cytometry

Plots

Confirm that:

- [x] The axis labels state the marker and fluorochrome used (e.g. CD4-FITC).
- [x] The axis scales are clearly visible. Include numbers along axes only for bottom left plot of group (a 'group' is an analysis of identical markers).
- [x] All plots are contour plots with outliers or pseudocolor plots.
- [x] A numerical value for number of cells or percentage (with statistics) is provided.

Methodology

Sample preparation	Delineated in the Methods.
Instrument	BD LSRII and BD LSR Fortessa flow cytometers (Becton, Dickinson and Company, Franklin Lakes, New Jersey, United States)
Software	BD FACSDiva v8.0.1 for collection, and FlowJo v10.7.1 for analysis.
Cell population abundance	Prevalence of populations are delineated in the plots and/or text.
Gating strategy	Figures depicting the gating strategies are supplied in the Supplementary Information. Gates were set using fluorescence minus one (FMO) controls where applicable.

- [x] Tick this box to confirm that a figure exemplifying the gating strategy is provided in the Supplementary Information.
